# Agility training to integratively promote neuromuscular, cardiorespiratory and cognitive function in healthy older adults: a one-year randomized-controlled trial

**DOI:** 10.1186/s11556-023-00331-6

**Published:** 2023-11-11

**Authors:** Eric Lichtenstein, Steffen Held, Ludwig Rappelt, Jonas Zacher, Angi Eibl, Sebastian Ludyga, Oliver Faude, Lars Donath

**Affiliations:** 1 Department of Sport, Exercise and Health, University of Basel, Grosse Allee 6, Basel, 4052 Switzerland; 2 Department of Intervention Research in Exercise Training, Institute of Exercise Training and Sport Informatics, German Sport University Cologne, Am Sportpark Muengersdorf 6, Cologne, 50933 Germany; 3 Institute of Cardiology and Sports Medicine, German Sport University Cologne, Am Sportpark Muengersdorf 6, Cologne, 50933 Germany

**Keywords:** older adults, agility training, strength, balance, cognition, multicomponent exercise, fall prevention

## Abstract

**Background:**

Exercise training recommendations for seniors include the targeted training of strength, balance, endurance and flexibility domains. Agility training (AT) is conceptualized as a multi-component and time-efficient training framework for older adults to improve physical, functional and cognitive health domains that are relevant for maintaining activities of daily living. The aim of this one-year trial was to comparatively evaluate the effects of agility training on physical and cognitive function.

**Methods:**

Seventy-nine healthy older adults (AT: 61.5% female, 70.8 ± 4.8 years, 27.7 ± 4.2 kg/m^2^; CG: 60.5% female, 69.6 ± 4.7 years, 27.5 ± 4.4 kg/m^2^) took part in this one-year randomized controlled intervention and were either assigned to the agility training group (AT) with two weekly 60 min AT sessions or to the control group (CG), receiving no treatment. Participants were assessed pre, intermediate and post intervention for strength and power, balance, gait speed under multi-task conditions, aerobic capacity as well as cognitive performance. Linear mixed effects models were used to analyze the effect of treatment over time.

**Results:**

Fifty-four participants (AG: 25, CG: 29) were analyzed, most drop-outs attributed to COVID-19 (17/30 dropouts). Adherence was good (75%) of 90 offered sessions. Notable effects in favor of AT were found for gait parameters in single (d = 0.355, Δ = 4.3%), dual (d = 0.375, Δ = 6.1%) and triple (d = 0.376, Δ = 6.4%) task conditions, counter movement jump performance (strength and power) (d = 0.203, Δ = 6.9%), static one leg balance (d = 0.256, Δ = 12.33%) and n-back reaction time (cognitive performance) (d = 0.204, Δ = 3.8%). No effects were found for the remaining outcomes (d < 0.175).

**Conclusion:**

AT might serve as an integrative training approach for older adults particularly improving gait and lower limb power parameters. It seems suitable to improve a broad range of seniors’ health domains and should replace isolated training of these domains. However, individual variation and progression of exercises should be considered when programming agility training providing adequate challenges throughout a long-term intervention for all participants.

**Trial registration:**

DRKS, DRKS00017469. Registered 19 June 2019—Retrospectively registered.

**Supplementary Information:**

The online version contains supplementary material available at 10.1186/s11556-023-00331-6.

## Key messages

- This is the first long-term randomized controlled trial that evaluates the efficacy of an agility training for older adults with regard to different neuromuscular, cognitive and cardiovascular performance measures.

- Agility training has a high adherence rate of 75% and a low drop-out rate (16.5%) over a whole year.

- Small effects in favor of the agility training group were revealed for functional neuromuscular performance but not for maximum strength, dynamic balance, cardiovascular capacity, or cognitive function.

- Future studies should include higher and progressive loads/intensities or challenging tasks to foster neuromuscular, cognitive and cardiovascular adaptations when training healthy older adults.

**Supplementary Information:**

The online version contains supplementary material available at 10.1186/s11556-023-00331-6.

## Background

Healthy and successful aging from an individual and societal perspective includes physical, cognitive, social and psychological factors [1]. Regular physical activity (PA) that fulfill the PA recommendations [2] reduces the risk for numerous non-communicable diseases and helps to maintain an independent lifestyle [3]. Available exercise training guidelines for older adults separately cover strength and balance to positively influence fall risk, endurance to positively affect cardiovascular health and flexibility in order to maintain mobility [2, 4, 5]. Up to 300 min of aerobic physical activity is recommended for optimal health benefits and it is suggested that older adults also perform at least two sessions of strength training and at least three sessions of functional balance and flexibility training per week [2]. As a consequence, Donath and colleagues [6] developed and proposed a multi-component and time-efficient agility training framework for older adults that involves accelerations and decelerations, changes of direction, strength and balance tasks as well as cognitive challenges, mirroring relevant functional demands of activities of daily living (ADLs). This approach aims to provide individual challenges with increasingly difficult and complex exercises. In this context, multi-component and group-based exercise training approaches with older adults revealed relevant improvements in strength [7–9], balance [10, 11], cognition [12], endurance [8, 10], and functional mobility [8, 9, 13]. However, these exercise interventions trained these components serially rather than in parallel limiting the potential to elicit physical and also cognitive benefits. Also, during daily life situations that require these components to function properly in order to avoid falls, no total focus on these components is usually present which is in stark contrast to the mentioned exercise interventions. Agility training attempts to divert or divide focus on a single component and thus potentially reflecting those situations more closely.

One pilot study implemented a training program following the agility framework proposed by Donath and coworkers [6] with older adults for eight weeks and compared agility training to a traditional strength and balance training program [14]. They observed larger improvements in endurance, balance and ankle strength in the agility training group compared to control group. However, long-term interventions, that have been shown to yield superior effects to short-term interventions for fall prevention [15] and balance performance [16], using a randomized-controlled study design utilizing the agility framework are missing. Long-term multi-component training with some minor agility-based aspects already improved walking speed, strength, self-rated physical functioning and dynamic balance [7, 17], all of which are measures attempting to gauge fall risk.Against this background, the aim of this one-year randomized controlled trial was to evaluate the effects of agility training on a broad array of neuromuscular, cardiovascular and cognitive performance parameters in community-dwelling, healthy older adults [18]. Those parameters’ associations with either fall risk or mortality and their potential changes due to training would allow us to draw conclusion whether the agility-training is an effective prevention strategy that could be included into the physical activity guidelines for elderly adults reducing their overall recommended training volume and this potentially improving compliance to the guidelines. Measures included, among others, the counter movement jump, chosen for its potential relation to fall risk [19] and its representation of the power capabilities of the power limbs that have been deemed more important than maximum isometric strength for the avoidance of falls. We specifically hypothesized that the training induces improvements with at least moderate effect sizes in all performance measures after the one-year intervention period when compared to a control group. This would strongly support the inclusion of agility-based exercises in older adults’ physical activity guidelines.

## Methods

### Study design

The study was designed and conducted as a one-year, parallel group, randomized controlled intervention trial. A study protocol with a detailed description of all methodological aspects has been published previously [18]. 5 by 5 block randomization utilizing the minimization method was applied to stratify participants to either the agility training group (AT) or the control group (CG) based on age, sex, BMI, maximum knee extension strength, dual task gait speed and VO_2_peak [20] by a researcher otherwise not part of the execution of the study. Couples were stratified to the same group due to infrastructural, motivational and interference issues. The number of couples was evenly balanced in both groups.

Recruitment was done by newspaper advertisement in 2018, and the intervention ran from February of 2019 up to March of 2020 in a large western European urban area. Participants had to be above 60 years of age, healthy, retired, and living independently in the community. They had to provide clearance from their general practitioner and were excluded if one of the following conditions was present: more than two structured training sessions per week within the last three months, travel time exceeding two months during the study, heavy smoking, BMI above 35 kg/m^2^, mini-mental state examination score below 26. Participants presenting with contraindications for exercise training were also excluded.

The CG received brief written information on the relevance and volume of health-related physical activity prior to the start of the intervention and no further treatment. They were further instructed to maintain their habitual physical activity behavior. Both groups were asked to keep a physical activity diary during the one-year period. All moderate to strenuous (metabolic equivalent (MET) ≥ 3.3) physical activities were calculated as MET-scores [21] and their durations were summed up to calculate their monthly physical activity levels. Physical activity was divided into tertials for analysis in order to attenuate outliers in physical activity reporting. All primary and secondary outcomes (Table 1) were collected before (T1) and after (T3) the intervention. After six months (T2 at midpoint of the one-year intervention) only selected measurements were collected due to economic constraints.

**Table 1:** Outcome measures

Outcome	Tool	Unit
Primary Endpoints		
Maximum knee extension strength	Maximum and relative isometric knee extension strenght in seated leg extension machine (Edition-Line, gym80, Gelsenkirchen, Germany) with force transducer (mechaTronic, Hamm, Germany)	[N] and [N/kg]
Reactive balance performance, eyes open	Postural sway upon perturbation on two-dimensional platform (Posturomed, Haider Bioswing, Pullenreuther, Germany) with acceleration sensor (MicroSwing®6, Haider Bioswing, Pullenreuth, Germany)	[mm]
Dual task gait speed	8m habitual gait speed while counting backwards in steps of three, measured with photoelectric time gates (DLS/F03, Sportronic, Leutenbach-Nellmersbach, Germany)	[m/s]
Agility^a^	Total and split times of the ACE measured with photoelectric time gates (DLS/F03, Sportronic, Leutenbach-Nellmersbach, Germany)	[s]
Secondary Endpoints		
Neuromuscular		
Maximum leg curl strength	Maximum and relative isometric leg curls strenght in prone leg curl machine (Edition-Line, gym80, Gelsenkirchen, Germany) with force transducer (mechaTronic, Hamm, Germany)	[N] and [N/kg]
Maximum handgrip strength	Maximum and relative isometric hangrip strength in standing upright position measured with hand dynamometer (Digimax, Hamm, Germany) with force transducer (mechaTronic, Hamm, Germany)	[N] and [N/kg]
Explosive strength of lower extremeties	CMJ height on force plate (FP4060-15 Bertec, Columbus, OH, USA)	[m]
Reactive balance performance, eyes closed	Postural sway upon perturbation on two-dimensional platform (Posturomed, Haider Bioswing, Pullenreuther, Germany) with acceleration sensor (MicroSwing®6, Haider Bioswing, Pullenreuth, Germany)	[mm]
Static balance performance, higher support	Total sway of the COP in tandem stance on force plate (FP4060-15 Bertec, Columbus, OH, USA)	[m]
Static balance performance, lower support	Total sway of the center of pressure in one-legged stance (COP) on force plate (FP4060-15 Bertec, Columbus, OH, USA)	[m]
Gait speed	8m habitual gait speed measured with photoelectric time gates (DLS/F03, Sportronic, Leutenbach-Nellmersbach, Germany)	[m/s]
Triple task gait speed	8m habitual gait speed while counting backwards in steps of three and simultaneously carrying a glass filled with water (to ¾), measured with photoelectric time gates (DLS/F03, Sportronic, Leutenbach-Nellmersbach, Germany)	[m/s]
Cardiovasular		
Aerobic capacity^a^	VO2max(rel) (Cortex Metamax, Leipzig, Germany) during spiroergometric test on a cycle ergometer (Ergoline, Bitz, Germany)	[ml/min/kg]
Cognitiv		
Inhibitory control^a^	Eriksen-Flanker task accuracy (congruent and incongruent trials) and reaction time (congruent and incongruent trials)	[% correct] and [ms]
Working memory^a^	N-back task Hit rate and reaction time (target and non-target)	[% correct targets/error rate] and [ms]

*ACE* Agility Challenge for the Elderly, *CMJ* Counter Movement Jump, *COP* Center of Pressure; VO2max(rel) Relative maximal oxygen consumption

^a^these measures were not part of the interim-assessment

### Outcomes measures

According to the main components of the agility training approach, we selected neuromuscular measures of strength, balance and measures of cardiovascular and cognitive performance as outcomes. Table 1 presents all included primary and secondary outcomes. A detailed description of all measurement procedures and data processing is available in the study protocol [18]. Cardiovascular parameters were assessed on a day before the assessment of neuromuscular and cognitive parameters with at least two days between the appointments. The time of day of the measurements was matched as good as possible (within three hours) between the timepoints.

### Intervention

Participants of the AT trained twice a week in three separate groups with a maximum of 13 participants in each group, supervised by two trained student assistants with a background in sports science (at least bachelors’ degree) that were specifically educated on agility-training. One session lasted 60 min, divided by a 10 min agility-specific warm-up, 45–50 min of agility training and a 5 min cool-down. For a progressive course of training, the one-year intervention period was divided into thirds (eFigure 1). A detailed description of the intervention can be found in Morat et al. [18]. The coaches documented adherence for every training session. During the second half of the one-year training period, four randomly chosen participants wore heart rate (HR) sensors (Polar, H7, Buettelborn, Germany) in each session to exemplarily capture and monitor cardiovascular training intensity. Mean average HR (HRavg) and mean peak HR (HRpeak) relative to participants mean maximum HR (HRmax) that was achieved during the spiroergometric test were calculated for each session.

### Statistics

Linear mixed effects models with random slopes and intercepts per participant were used to investigate differences in performance trajectories between groups the three time points [22]. Therefore, a time by group interaction model was used. In the default model (Model 1), only this interaction was included and in a second model (Model 2) with sex,BMI and physical activity as covariates. The estimate of the time by group interaction can be interpreted as the difference between the group deltas. Effect sizes were standardized to the baseline standard deviation of the respective parameter. Effect sizes are interpreted as trivial (< 0.2), small (0.2–0.49), moderate (0.5–0.79), large (0.8–1.19) and very large (> 1.2). Descriptive data is presented as means and standard deviations, differences as means or effect sizes with 95% confidence intervals. Participants that dropped out of the study were excluded from the analysis but were compared to the participants that did not drop out to detect possible drop-out bias. Data were analyzed using an intention-to-treat approach and missing data were handled by utilizing the mixed effects model that is robust against missing data [23]. Statistical analyses were performed with R (Version 4.1.1, R Foundation) utilizing the package lme4 (Version 1.1–27.1).

## Results

### Study sample

**Fig. 1:**
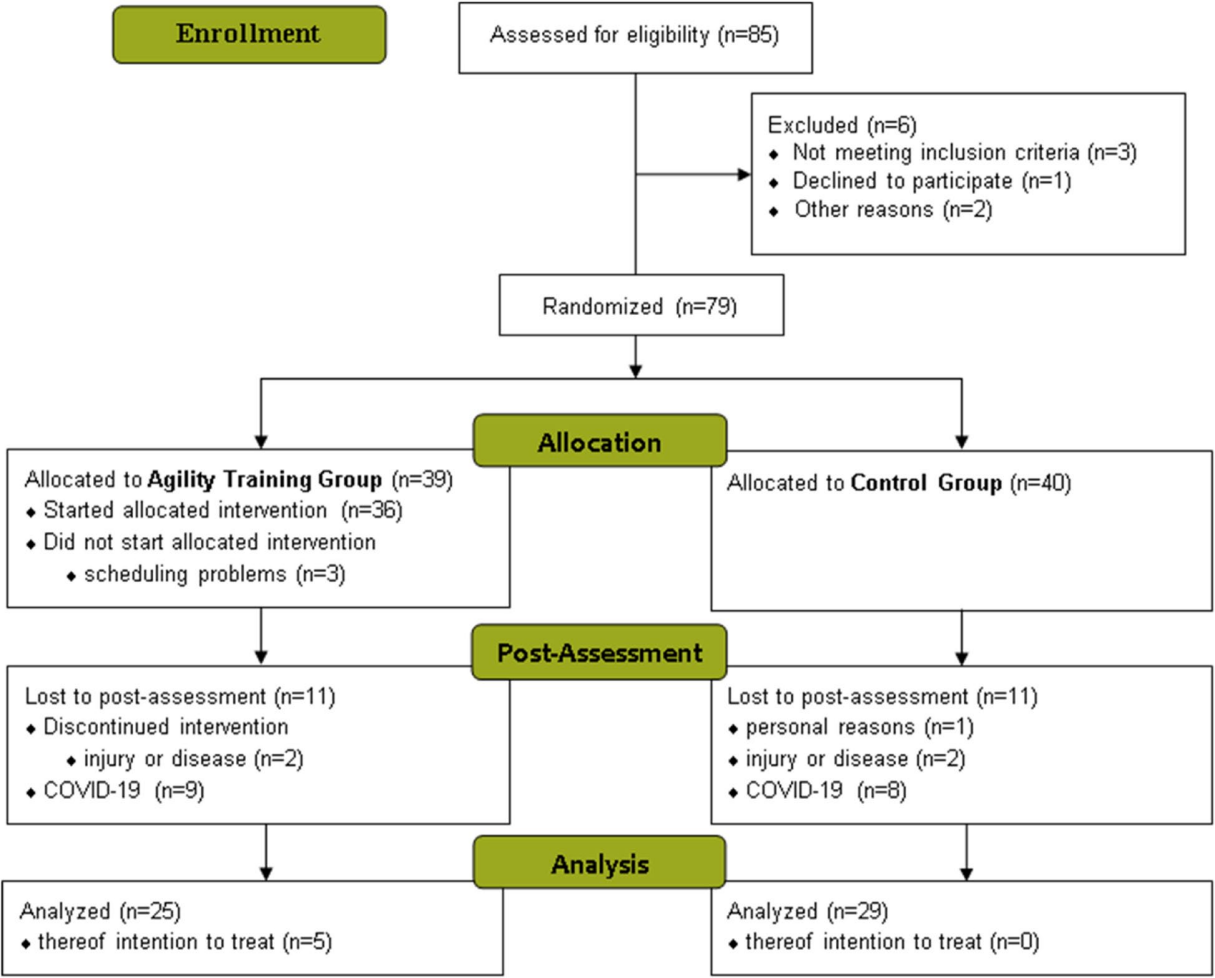
Participant flow

Seventy-nine participants were randomized to the intervention arms, 76 started the intervention as allocated and 22 participants were lost to the post-assessment (Fig. 1). Of 39 participants in the AT, 36 started the intervention as allocated and 25 were available for post-assessment, including five that discontinued the intervention. In the CG, 29 out of 40 randomized participants were present at post-assessment. The greatest loss of participants was due to COVID-19, which caused a sudden end of the study during post-assessment. The dropout rate between randomization and post-assessment was 16.5% (25.6% AT, 7.5% CG) without COVID-19 dropouts and 38% (48.7% AT, 27.5% CG) with COVID-19 dropouts.

Baseline characteristics of the participants who were part of the analysis are listed in Table 2. Participants that dropped out (unrelated to COVID-19) did not differ from the participants that did not drop out. However, including participants that dropped out due to COVID-19 potential differences were observed. They had on average less endurance capacity (VO_2_max: -7.0%), weaker leg strength (extension: -7.2%, flexion: -9.9%,), and less leg power (-6.3%) than the ones finishing the study.

**Table 2:** Baseline characteristics of participants who were part of the analysis

Characteristic	AT	CG	Total (*n* = 54)
Gender, female/male (n)	15/10	18/11	33/21
Age [years]	70.8 (4.8)	69.6 (4.7)	70.2 (4.7)
Weight [kg]	78.3 (14.9)	79.3 (15.3)	78.8 (15.0)
Height [m]	1.68 (0.09)	1.70 (0.1)	1.69 (0.10)
BMI [kg/m^2^]	27.7 (4.2)	27.5 (4.4)	27.6 (4.2)
MMSE [score]	28.6 (1.1)	28.5 (1.1)	28.6 (1.1)
VO_2_max [ml/min/kg]	24.3 (4.9)	25.5 (6.4)	24.9 (5.7)
Physical activity [min/week]	544 (325)	604 (456)	575 (396)

Data are mean and standard deviation unless noted otherwise

*AT* Agility training group, *CG* Control Group, *MMSE* Mini Mental State Examination

### Training adherence and intensity

Adherence of the AT was 75.0% (70 (range: 50–86) of 90 (83–95) training sessions). The time, spent in different agility domains throughout the thirds of the intervention period and for every single training session is displayed in eFigures 2 and 3. Within AT, the compliance was not associated with changes over time for all parameters, except for reactive balance with open eyes, where a 1% increase in compliance was associated with a sway path reduction of 0.64 to 7.51 mm. Mean HRavg and mean HRpeak of all evaluated training sessions were 71.2% (SD: 3.2) of HRmax and 92.5% (5.2) of HRmax respectively. Apart from selective and rare post exercise muscle soreness, no harmful effects were reported during the intervention.

### Outcome measures

**Fig. 2:**
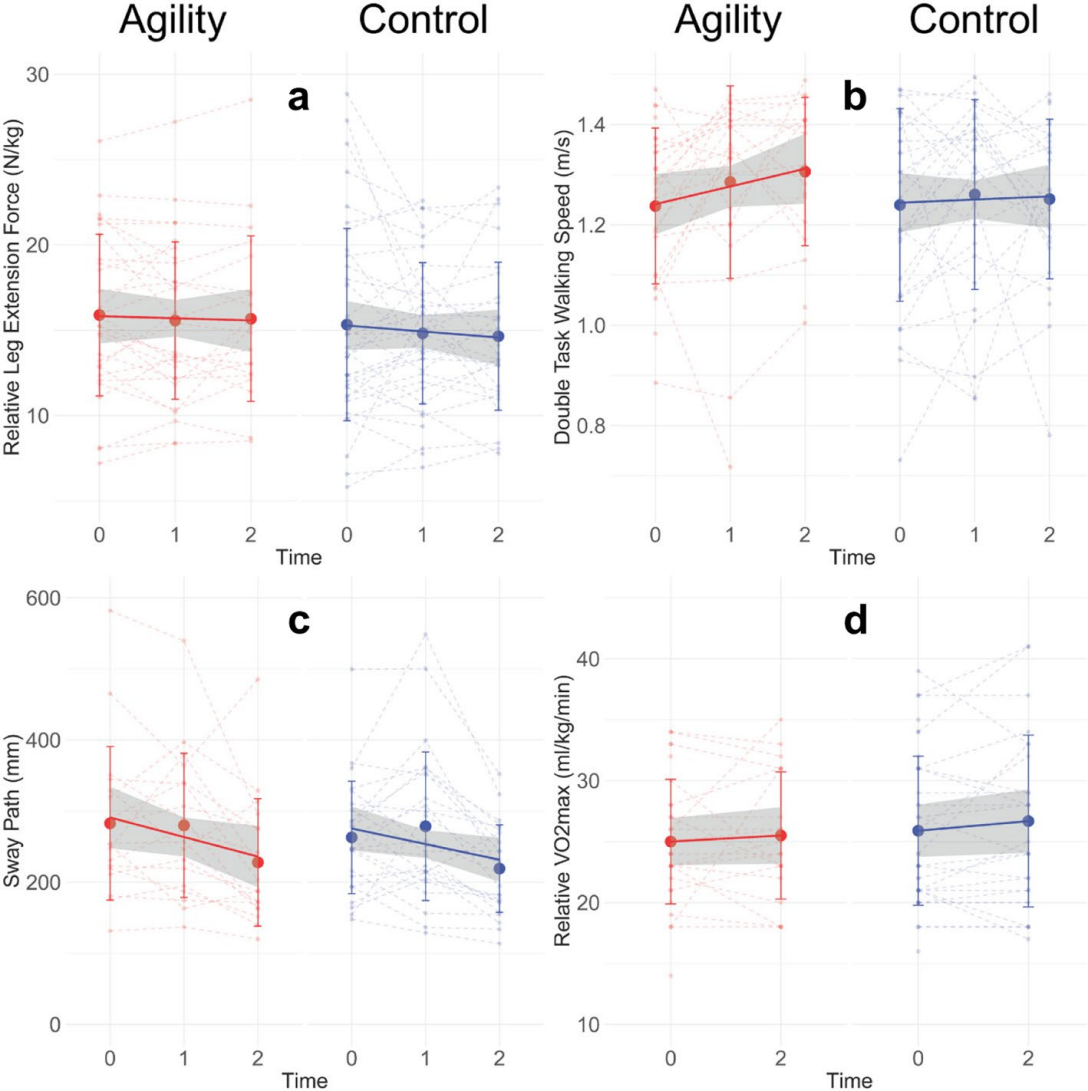
Visualisation of individual (dashed) and group specific average performance trajectories for relative knee extensor strength (**a**), dual task walking speed (**b**), dynamic balance with open eyes (**c**) and endurance capacity (**d**). Error bars indicate standard deviations and grey area 95% confidence interval of the group trajectory

Numbers of valid measures, mean values and standard deviation for AT and CG, as well as the results of both mixed models are summarized in Table 3. The number of valid measures differs from the total number of analyses for each outcome due to errors in measuring procedures, participants’ inability to perform measurement, non-valid trial and the exclusion of outliers. Small effect sizes in favor of the AT were found for gait speed in all conditions (0.355 < d < 0.376), static one-leg balance (d = 0.256), CMJ performance (d = 0.203), flanker reaction time to incongruent stimuli (d = 0.203) and Nback reaction time on correct targets (d = 0.205). A small effect size in favor of the CG was found for flanker accuracy to incongruent stimuli (d = 0.246), which in combination with the reduced reaction time represents a speed-accuracy trade-off and, therefore, no improvement in that task. For gait parameters and CMJ performance, the data is also compatible with trivial to moderate effects, whereas for the other parameters, moderately negative to large positive effects are compatible with the data. Figure 2 visualizes the different trajectories for the two groups over time for the three main outcomes and a cardiovascular parameter.

**Table 3:** Outcome measures with linear mixed effects comparison

		AT				CG				Effect size for Group*Time Interaction in favor of AT			
Outcome		n	Pre mean (SD)	Interim mean (SD)	Post mean (SD)	n	Pre mean (SD)	Interim mean (SD)	Post mean (SD)	Effect Model 1	95% CI	Effect Model 2	95% CI
Gait	single [m/s]	19	**1.37 (0.18)**	**1.4 (0.15)**	**1.38(0.13)**	27	**1.4 (0.16)**	**1.42 (0.14)**	**1.36 (0.12)**	**0.355**	[-0.09;0.8]	**0.241**	[-0.22;0.7]
	dual taks [m/s]	20	**1.27 (0.19)**	**1.33 (0.14)**	**1.35 (0.15)**	27	**1.27 (0.02)**	**1.36 (0.23)**	**1.29 (0.18)**	**0.375**	[-0.1;0.85]	**0.469**	[0.07;0.87]
	triple task [m/s]	20	**1.26 (0.18)**	**1.33 (0.15)**	**1.33 (0.14)**	27	**1.25 (0.19)**	**1.35 (0.2)**	**1.25 (0.18)**	**0.376**	[-0.01;0.76]	**0.378**	[0;0.76]
Balance	static one-legged [m]	19	**0.57 (0.25)**	**0.50 (0.22)**	**0.48 (0.23)**	23	**0.45 (0.12)**	**0.47 (0.15)**	**0.41 (0.10)**	**0.256**	[-0.61;1.12]	0.181	[-0.16;0.52]
	static tandem [m]	21	0.35 (0.17)	0.37 (0.16)^c^	0.31 (0.16)	26	0.34 (0.11)	0.33 (0.07)	0.32 (0.11)	0.010	[-0.31;0.33]	0.131	[-0.21;0.47]
	dynamic open eyes [mm]	20	287 (105)	284 (98)	234 (89)	25	263 (79)	279 (104)	219 (61)	0.120	[-0.59;0.83]	0.144	[-0.35;0.63]
	dynamic closed eyes [mm]	18	448 (305)	362 (160)^c^	341 (254)	24	365 (193)	389 (232)	273 (142)	0.099	[-0.52;0.72]	0.175	[-0.24;0.59]
Strength	Counter Movement Jump [m]	19	**0.13 (0.06)**	**0.13 (0.05)**	**0.14 (0.06)**	22	**0.14 (0.04)**	**0.13 (0.04)**	**0.14 (0.04)**	**0.203**	[-0.13;0.53]	0.122	[-0.14;0.39]
	Frel leg extension [N/kg]	18	14.8 (5.2)	14.8 (5.2)	15.2 (5.5)	24	14.7 (5.4)	14.8 (4.0)	14.8 (4.6)	0.082	[-0.21;0.37]	-0.001	[-0.27;0.27]
	Frel leg curl [N/kg]	17	6.6 (3.0)	6.8 (2.7)^c^	7.2 (2.5)	24	6.3 (2.2)	6.4 (2.1)	6.5 (1.9)	0.146	[-0.06;0.35]	0.164	[-0.11;0.44]
	Fmax handgrip [N]	20	325 (101)	323 (89)	319 (94)	25	302 (95)	307 (100)	301 (101)	-0.006	[-0.21;0.19]	0.06	[-0.2;0.32]
	Frel handgrip [N/kg]	20	4.1 (1.2)	4.1 (1.1)	4.1 (1.2)	25	4.0 (1.1)	4.1 (1.1)	4.0 (1.2)	0.081	[-0.18;0.34]	0.059	[-0.25;0.37]
Cognition	Flanker accuracy congruent [%]	24	0.97 (0.04)	-	0.97 (0.03)	29	0.96 (0.04)	-	0.96 (0.05)	-0.022	[-0.62;0.58]	**-0.552**	[-1.19;0.08]
	Flanker accuracy incongruent [%]	25	**0.92 (0.08)**	**-**	**0.91 (0.07)**	29	**0.90 (0.08)**	**-**	**0.91 (0.09)**	**-0.246**	[-0.75;0.26]	**-0.453**	[-1.04;0.14]
	Flanker reaction time congruent [ms]	24	442 (63)	-	431 (50)	28	421 (39)	-	416 (36)	0.106	[-0.33; 0.54]	0.053	[-0.48;0.59]
	Flanker reaction time incongruent [ms]	24	**469 (69)**	**-**	**455 (54)**	28	**442 (43)**	**-**	**440 (39)**	**0.203**	[-0.22; 0.62]	**0.201**	[-0.32;0.73]
	Nback hit-rate [%]	25	0.85 (0.12)	-	0.89 (0.07)	29	0.84 (0.19)	-	0.86 (0.14)	0.175	[-0.32;0.67]	**0.379**	[-0.19;0.95]
	Nback reaction time target [ms]	24	**621 (121)**	**-**	**576 (115)**	29	**604 (111)**	**-**	**582 (114)**	**0.205**	[-0.14; 0.56]	0.16	[-0.24;0.56]
	Nback reaction time non-target [ms]	24	675 (140)	-	646 (134)	29	665 (158)	-	650 (170)	0.091	[-0.21; 0.40]	-0.012	[-0.37;0.35]
Endurance	VO2max rel [l/min/kg]	25	25.0 (5.2)	-	24.9 (5.6)	25	26 (6.2)	-	26.7 (7.0)	-0.142	[-0.51;0.22]	**-0.266**	[-0.73;0.2]

Number of valid measures (n); mean (SD = standard deviation) and linear mixed effect model results for the interaction as effect size. Lines printed bold show at least small effects (> 0.2)

Model 1: univariate time*group model with random slope and intercept for individuals

Model 2: Model 1 with sex, BMI, and time*MET_cat as covariates

Average activity levels of the AT were 5664 ± 2415 METmin/month including agility training sessions. The CG achieved 5900 ± 3222 METmin/month with no difference between groups.

Due to Covid-19 and the measuring schedule, only 20 participants (all AT) were assessed for ACE performance. All split times and the total time to finish the course decreased from T1 to T3 (eTable 1). Accounting for excellent reliability (ICC = 0.93) [24], a sequential data analysis without control seems justified. Furthermore, participant’s improvements in total time were 8.6% and thus distinctly exceeded absolute variability of the test (CV = 4%) [24].

## Discussion

This study evaluated the effects of a two-armed one-year randomized controlled trial applying the agility training approach on neuromuscular, cognitive and cardiovascular performance in healthy older adults. Adherence was good for a long-term intervention study as an average adherence of 70% could have been expected from the analysis of McPhate and colleagues [25]. It was hypothesized that improvements in favor of the AT would occur in all performance measures. Our results merely confirm this hypothesis for selected parameters: Healthy and comparatively active older adults that regularly performed agility training notably improved their gait speed under all conditions as well as their lower limb power, static balance performance, and working memory compared to the control group, that only received standard physical activity recommendations over a one-year period. These findings appear relevant and plausible as moving through an environment full of distractions and unforeseen situations that should be reacted upon quickly, might be relevant in the context of fall prevention [26]. Surprisingly however, dynamic balance, maximum strength, and endurance performance parameters’ trajectories did not differ between groups. As the investigated population presented as highly active and very healthy this should, however, not be generalized to the whole population of older adults.

Other one-year studies applying multi-component exercise training [7, 17] that did not follow the agility framework observed improvements in force generating ability, dynamic balance and walking speed. Similar improvements were not observed in our study. In order to improve isolated strength and power performance, a minimum level of intensity (resistance or speed) seems required [27]. This minimal level might not be attained by mere walking-based exercises in very healthy and active older adults. The higher reported physical activity of the control group participants could be one possible explanation for this lack of findings. Future studies should therefore include high speed walking or multi-task resistances exercises with a load and load progression suitable for long term strength adaptations. However, a focus on isolated strength training to improve these proposed fall risk factors in an intervention seems unwarranted, as the link between maximum strength gains and performance gains in functional tasks, activities of daily living and fall rates can be considered rather weak given the current literature [28].

The slightly improved counter movement jump performance as a measure of explosive strength in this study, with an absence of meaningful improvement in isometric knee extension and flexion strength, might indicate improved reactive strength. This could be explained by the plyometric-style movements like cutting, stop and go and changes of directions that are at the core of the agility training program. This might be especially relevant for activities of daily living, where isolated concentric movements are rare and the ability to quickly react to perturbations and produce force fast is most relevant to avoid falling [29]. The usefulness of hand grip strength measurements to document intervention changes has recently received scrutiny, and a lack of intervention effects in this parameter are therefore not unsurprising especially in a highly active and healthy population [30]. Although standardized practice trials were performed, high learning effects are likely to occur in balance testing and a high baseline-level might have caused a ceiling effect of results [31, 32].

Englund and colleagues [17] found improvements in maximum walking speed of 11.4%, while in this study the agility training group maintained their performance compared to the control group that got 2.9% slower but improvements in the multi-task conditions were in the range of 5 to 6%. The discrepancy between the studies might be explained by Englund et al. [17] assessing the maximum walking speed while self-selected walking speed was investigated in this study and the different population that consisted of only women. Englund et al. [17] also measured habitual walking speed, but did not provide the corresponding data. How a 5% improvement in habitual walking speed would transfer to maximum walking speed improvements remains speculative. The assessment of gait reserve (maximum vs habitual speed) could be an interesting parameter for further studies [33] as well as walking variability and the ability to adapt gait to external perturbations or unforeseen events.

No difference in the change of cardiorespiratory capacity was observed between the groups. A strong link between the maintenance of cardiovascular capacity and physical activity was reported [34]. As the physical activity level during the study intervention was quite similar in both groups (5664 vs 5900 METmin/month, *p* = 0.79), the lack of differences seems plausible. Interestingly, without the intervention training, AT would have had meaningful lower physical activity (3564 vs 5900 METmin/month, *p* < 0.05), supporting the assumption of a reduced leisure time physical activity level compensation for the intervention [35]. The recorded training intensity on a cardiovascular level was sufficient to usually lead to improvements in endurance performance [36], but at 70% HR_max_ average intensity, peripheral adaptations might be more likely than central adaptations [37]. The ergometer-based test would require specifically improved performance in the thigh musculature, whereas the training adaptations can be expected over many different muscle groups (e.g. calf and hip). Other studies utilizing similar intensities in the same population revealed improvements in endurance performance [38, 39], but also utilized more functional assessments such as the six-minute walking test, which seems more specific to assess walking-based endurance performance than a graded cycle ergometer test.

The performance parameters of the Eriksen-Flanker test were not positively influenced by the intervention, while agility training improved performance on the n-back test. This positive effect on working memory was indexed by a reduction of reaction time on targets at comparable accuracy. The high utilization of memory-based tasks within the intervention compared to reaction tasks might explain these divergent findings on cognitive abilities. Other studies have failed to improve cognitive function when combining cognitive training and physical activity [40, 41]. The present findings can be considered relevant, as working memory is required for learning and refining movements and adjusting them to new environments and situations.

The challenge in this setting arises to provide an adequate volume of cognitive training into the intervention. A low duration (< 30 min) of cognitive training sessions in computer-based interventions has been shown to have the least effect on cognitive function compared to longer durations [42]. The actual duration of cognitive training in interventions where physical exercises and cognitive training are paired is difficult to evaluate. Considering the present intervention design, challenging cognitive tasks where likely to short and infrequent to induce meaningful adaptations. Additionally, the interventions’ cognitive tasks were not calibrated to an individual’s capabilities and might have been to complex blocking adaptations. Further, cognitive tasks were not progressed throughout the intervention but remained largely similar.

Compared to normative data in this age group [43, 44] the participants of the study performed well for their age, potentially stunting intervention effects that could be observed in less fit participants. Participants were also highly active in both groups, more than doubling recommended doses of physical activity on average (5805 vs 2000 METmin/month) [35]. Nevertheless, the high drop out rate due to the Covid-19 pandemic severely reduced the power of the study and results should, therefore, be interpreted with caution. As a link between fitness and immune function exists [45, 46], a small survivorship bias is possible as participants finishing the study were potentially fitter than the drop-outs. As adaptations in less fit participants can be expected to be larger, this might have hampered our intervention effects.

## Conclusion

While earlier studies showed very promising results of a short-term agility intervention [14], the long-term effects observed in this study were less promising in a group of very active and healthy older adults. Still, agility-based exercise training might serve as an integrative multi-component training approach for older adults that particularly improves gait and lower limb power parameters while simultaneously improving some cognitive function. A lack of intensity and complexity progression in the long-term training process should be avoided in future studies and exercises should be designed to challenge every participant on their individual level. While this potentially increases resources for planning and is less possible in a usual group setting of around 15 participants, small groups might be preferable. The high adherence and low unforced drop-out rate shows, that this kind of multi-component exercise is feasible and long-term desirable by healthy older adults.

### Supplementary Information

**Additional file 1: eFigure 1.** Training design and progression (from Morat et al. 2020). **eFigure 2.** Proportions of the different agility components of the total amount of exercise for the thirds of the one-year training intervention. **eFigure 3.** Proportions of the different agility components of the total amount of exercise for every single training session of the one-year training intervention. **eTable 1. **Agility Challenge for the Elderly Results.

## Data Availability

The datasets analyzed during the current study available from the corresponding author on reasonable request.
