# Supplementary material for: Agility training to integratively promote neuromuscular, cardiorespiratory and cognitive function in healthy older adults: a one-year randomized-controlled trial
Source: Eur Rev Aging Phys Act. 2023 Nov 11;20:21. doi: 10.1186/s11556-023-00331-6 (PMC10638759; doi:10.1186/s11556-023-00331-6)
Supplement: Supplementary file 1 — Additional file 1: eFigure 1. Training design and progression (from Morat et al. 2020). eFigure 2. Proportions of the different agility components of the total amount of exercise for the thirds of the one-year training intervention. eFigure 3. Proportions of the different agility components of the total amount of exercise for every single training session of the one-year training intervention. eTable 1. Agility Challenge for the Elderly Results. [file 11556_2023_331_MOESM1_ESM.docx]

**Supplemental material**

**eFigures**


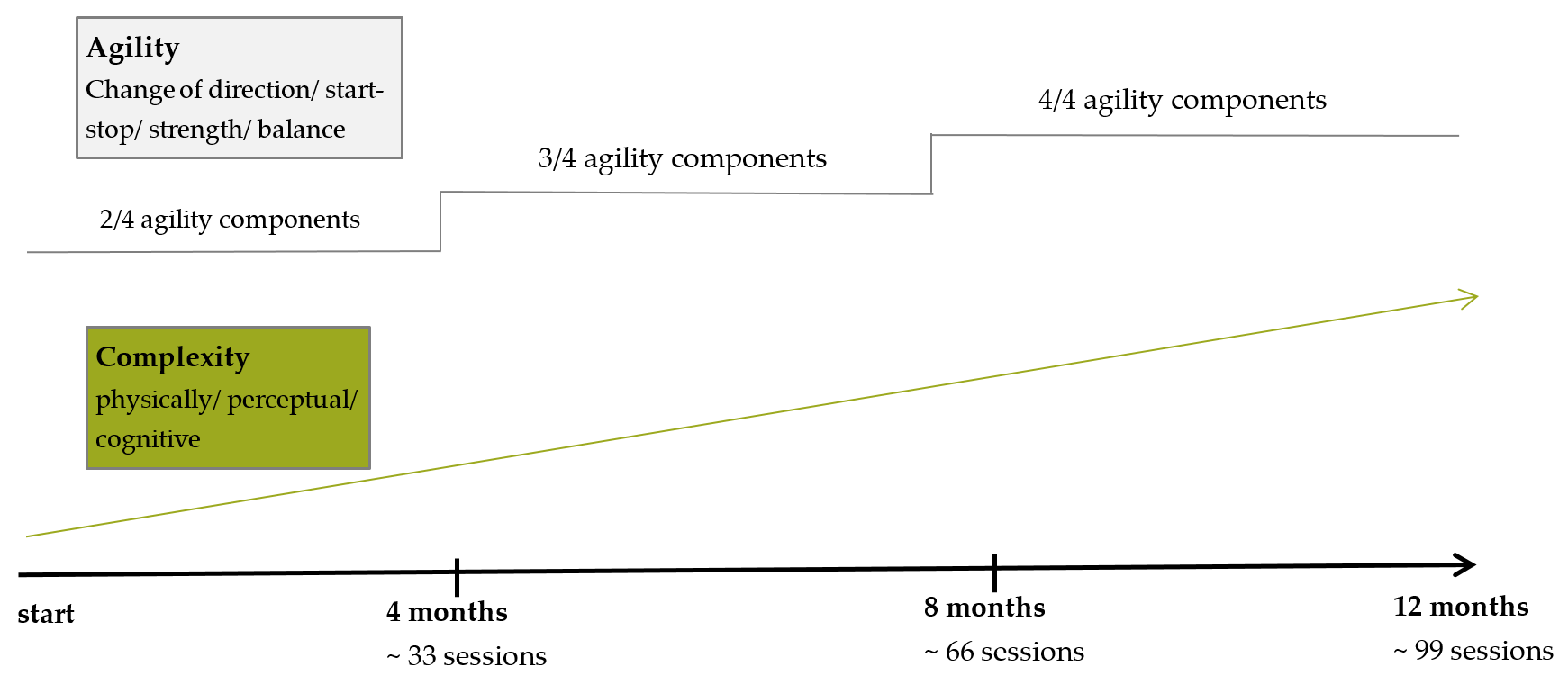


*eFigure 1* Training design and progression (from Morat et al. 2020)

Morat M, Faude O, Hanssen H, Ludyga S, Zacher J, Eibl A et al. Agility Training to Integratively Promote Neuromuscular, Cognitive, Cardiovascular and Psychosocial Function in Healthy Older Adults: A Study Protocol of a One-Year Randomized-Controlled Trial. *International journal of environmental research and public health*. 2020;17 (6).10.3390/ijerph17061853.


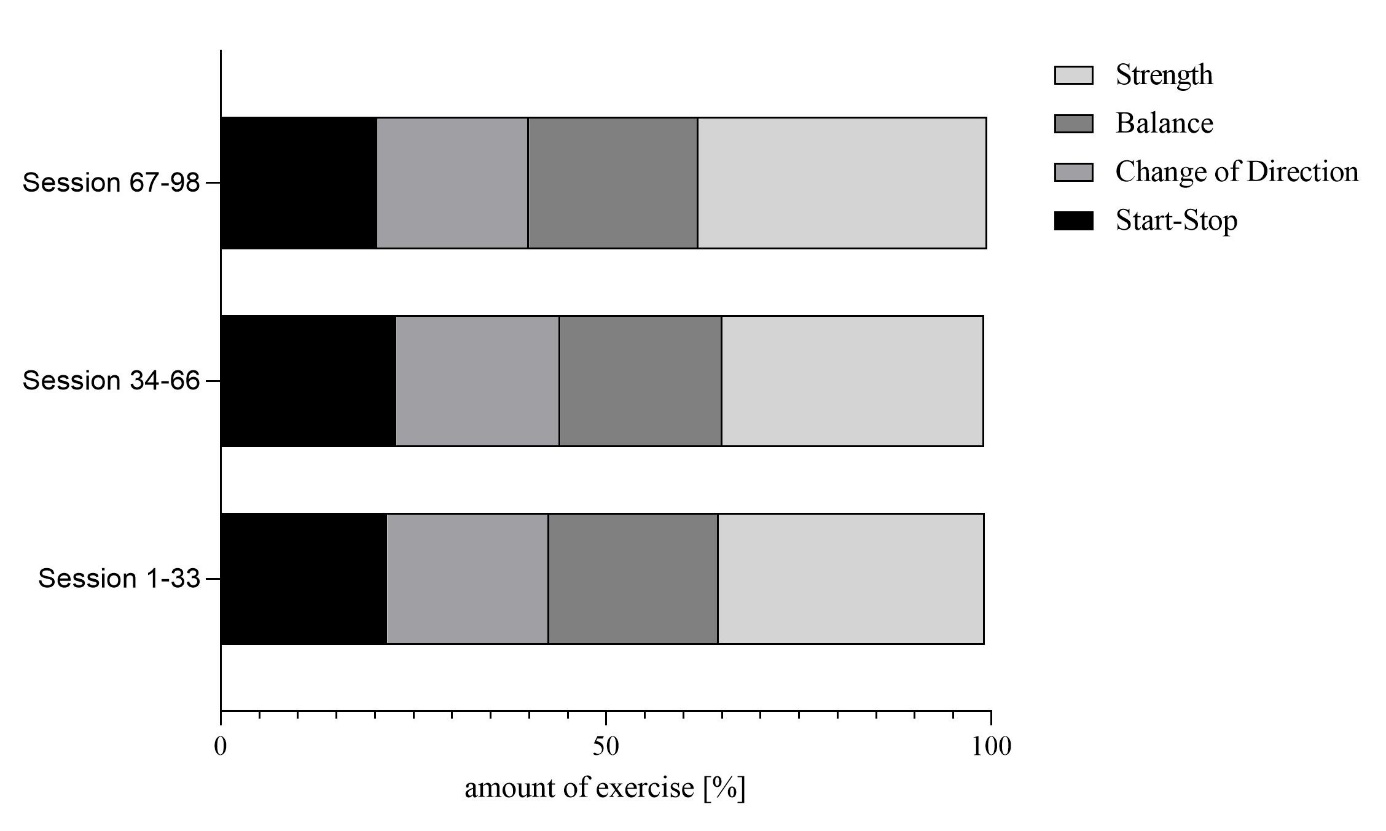


*eFigure 2* Proportions of the different agility components of the total amount of exercise for the thirds of the one-year training intervention


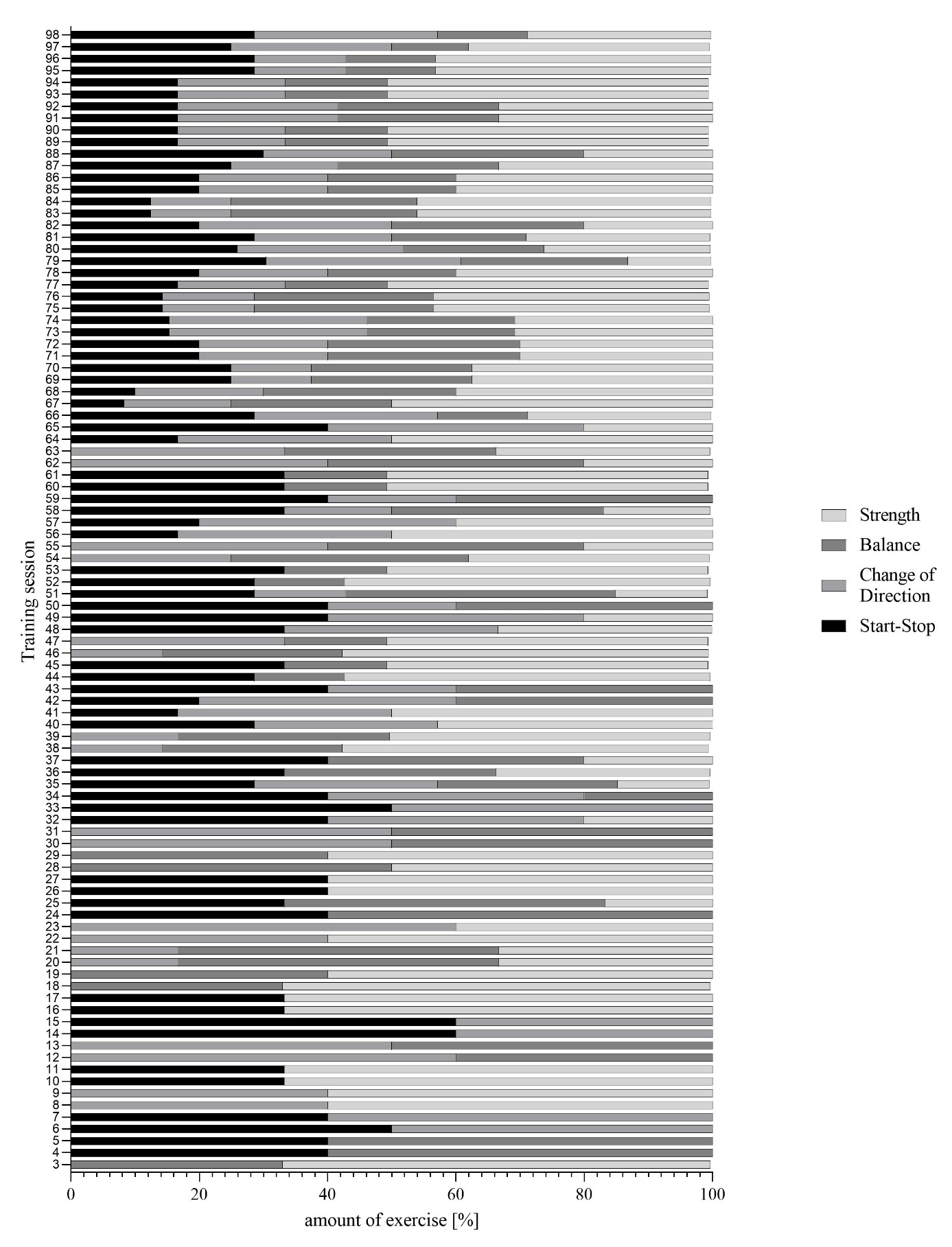


*eFigure 3* Proportions of the different agility components of the total amount of exercise for every single training session of the one-year training intervention

**eTables**

*eTable 1* **Agility Challenge for the Elderly Results**

| Outcomes | | n | pre  mean (SD) | post  mean (SD) | Δ (s) [95 % CI] | Δ (%) [95 % CI] |
| --- | --- | --- | --- | --- | --- | --- |
| Agility Challenge for the Elderly (ACE) | split time 1 [s] | 20 | 7.60 (1.19) | 6.68 (0.72) | -0.91 [-1.28; -0.55] | -12.0 [-16.8; -7.2] |
|  | split time 2 [s] | 20 | 14.85 (2.05) | 13.06 (1.36) | -1.79 [-2.42; -1.17] | -12.1 [-16.3; -7.9] |
|  | split time 3 [s] | 20 | 27.71 (3.75) | 26.38 (3.19) | -1.33 [-2.42; -0.24] | -4.8 [-8.7; -0.9] |
|  | total time [s] | 20 | 50.68 (6.83) | 46.31 (5.02) | -4.37 [-6.34; -2.40] | -8.6 [-12.5; -4.7] |
| Footnote: Number of valid measures (n); mean (SD = standard deviation) and t-test results for the Agility Challenge for the Elderly in the Agility training group (AT). CI = confidence interval. | | | | | | |
